# Psychological and environmental factors influencing resilience among Ukrainian refugees and internally displaced persons: a systematic review of coping strategies and risk and protective factors

**DOI:** 10.3389/fpsyg.2023.1266125

**Published:** 2023-10-09

**Authors:** Damiano Rizzi, Giulia Ciuffo, Marta Landoni, Matteo Mangiagalli, Chiara Ionio

**Affiliations:** ^1^Fondazione Soleterre Strategie di Pace ONLUS, Milan, Italy; ^2^Unità di Medicina d’Urgenza, Dipartimento di Medicina Interna, Fondazione IRCCS Policlinico San Matteo, Pavia, Italy; ^3^Unità di Ricerca sul Trauma, Dipartimento di Psicologia, Università Cattolica, Milan, Italy; ^4^CRIdee, Dipartimento di Psicologia, Università Cattolica, Milan, Italy; ^5^Dipartimento di Scienze Clinico-Chirurgiche, Diagnostiche e Pediatriche, University of Pavia, Pavia, Italy

**Keywords:** war, Ukraine, mental health, risk factors, protective factors, coping strategies, refugees, IDPs

## Abstract

**Background:**

There is much discussion in the literature about the link between traumatic events related to war and mental illness. However, in comparison, mental health has been more researched than protective factors such as coping methods, which are the primary factors to build resilience in these circumstances. This review examines the psychological and environmental elements that influence the resilience of Ukrainian refugees and IDPs by analyzing coping strategies and risk and protective factors.

**Methods:**

A literature search was conducted on PsycINFO, Pubmed, Scopus, and Science Direct, with 259 articles screened and 13 determined as eligible for inclusion. Inclusion criteria were: (1) studies on adult Ukrainian refugees and/or IDPs; (2) original, peer-reviewed studies; and (3) studies written in English or Italian language. Single-case reports and qualitative studies were excluded, as well as those studies written in any other language, and any studies for which the full-text version could not be obtained (i.e., conference abstracts). Two reviewers independently reviewed titles and abstracts, reviewed relevant articles’ full text, and extracted the data.

**Results:**

A diverse range of individual and socio-environmental risk and protective factors were identified, influencing the resilience of Ukrainian refugees and IDPs, as well as five main categories of coping strategies: emotion-focused strategies, problem-focused strategies, avoidance, faith-based strategies, and the ones based on sense of belonging.

**Discussion:**

War trauma and associated stressors can lead to distressing physical and psychological reactions, which persist even after leaving the war zone. Many individual and socio-environmental risk factors, such as mental disorders, financial security, having relatives wounded or displaced, and an unfamiliar environment could influence the risk and severity of psychological difficulties, emphasizing the importance of coping strategies, social connections, faith, and cultural resilience.

**Conclusion:**

This systematic review underscores the complex range of coping strategies and factors influencing the resilience of Ukrainian refugees and IDPs. Social connections and inclusive community interventions play vital roles in improving their psychological well-being, while longitudinal studies and culturally sensitive support are needed to address their unique challenges and strengths. Implementing collaborative care models can provide comprehensive support by integrating mental health services with primary healthcare and community-based organizations.

## 1. Introduction

### 1.1. Understanding war-related migration: impact on mental health

According to the definition of those who are either states or aspire to become states, “war should be understood as an actual, deliberate and widespread armed conflict between political communities” (Orend, 2008). Indeed, “War remains one of the most complicated and destructive human endeavors, whether viewed from a philosophical, sociological or legal standpoint” (Orend, 2008, p. 49).

The ongoing Russian invasion of Ukraine, which began on 24 February 2022, is causing the largest civilian refugee disaster in Europe since World War II and the first of its kind since the Yugoslav war in the 1990s. The war in Crimea and Eastern Ukraine goes back to 2014, and had already resulted in many deaths, large groups of internally displaced people, and significant psychosocial problems.

United Nations High Commissioner for Refugees estimates that in 2021 there were approximately 84 million displaced persons worldwide, of whom 26.6 million were refugees and 4.4 million were seeking asylum (United Nations High Commissioner for Refugees, 2022a). These numbers are the highest recorded in the last 20 years. In this regard, the conflict in Ukraine has led to both alarming numbers and a precarious scenario. As of 29 November 2022, more than 7.8 million people had left the country and 8 million were internally displaced (United Nations High Commissioner for Refugees, 2022b). According to the United Nations High Commissioner for Refugees (2022b), about 1.6 million Ukrainian refugees live in Poland, and many more are hosted in surrounding countries. Indeed, the war is having an impact not only on the Ukrainian population, but also on people in neighboring countries.

According to the literature on this topic, war-related trauma is a traumatic experience that not only directly exposes a person to violence and atrocities or poses a threat to life or health. As it is a negative life experience, one is not only directly exposed to war, but also indirectly, e.g., when seeing images of combat on television or social media (e.g., Miller and Rasmussen, 2010; Denov et al., 2019; Anjum et al., 2023). According to the concept of indirect exposure, people who are affected by war but do not live in an area of conflict can also experience negative mental health effects, such as the population of countries close to Ukraine as well as countries hosting refugees (Essau and Trommsdorff, 1996; Chudzicka-Czupała et al., 2023a).

Among the most vulnerable demographic groups among refugees are children (more than half of all Ukrainian children have had to leave home), women, the elderly, and the sick, who are unable to participate in the military response (Dobson, 2022; Hodes, 2022). According to Charlson et al. (2019), Javanbakht (2022), and Elvevåg and DeLisi (2022), these people are at very high risk of developing a range of mental health conditions, including post-traumatic stress disorder (PTSD), severe anxiety and depressive symptoms, and suicidal thoughts or behavior. Johnson et al. (2022) found that internal migrants (IDPs) had a high incidence of PTSD symptoms across all socio-demographic categories due to their high level of direct exposure to conflict-related traumatic events (65%), even though the fighting was still confined to the Donbass and Kharkiv regions. Similar findings were reported by Roberts et al. (2019), who conducted a cross-sectional survey of IDPs in the early stages of the war and found that 32, 22, and 17% of IDPs suffered from PTSD, depression, and anxiety, respectively. According to Cheung et al. (2019), 55% of IDPs in Ukraine were at risk of somatic problems.

### 1.2. Coping strategies among refugees and internally displaced persons

There is much discussion in the literature about the link between traumatic events related to war and mental illness (Porter and Haslam, 2005; Steel et al., 2009). However, mental health research has come under criticism for adopting a limited, Western conception of trauma and mental disorders, particularly post-traumatic stress disorder (PTSD), which ignores the cultural roots of mental health and suffering and the different ways in which people and their communities deal with conflict (Kienzler, 2008; Miller and Rasmussen, 2010; Seguin and Roberts, 2015). In comparison, mental health has been more researched than protective factors such as coping methods, which are the primary factors to build resilience in these circumstances. Indeed, the main goals for mental health and primary care providers for families of refugees and IDPs can be seen in the promotion of resilience, post-traumatic growth and the strengthening of protective factors. Although researchers acknowledge the harmful effects of acute stress and mental health issues, in recent years they have begun to adopt a resilience approach.

Ungar (2008, p.225) describes resilience as: “*In the context of exposure to significant adversity, whether psychological, environmental, or both, resilience is both the ability of individuals to find their way to health-promoting resources, including the ability to experience feelings of well-being, and a condition of the individual’s family, community, and culture to provide these health resources and experiences in culturally meaningful ways*.”

According to Folkman and Lazarus (1980, p. 223), coping is defined as “*the cognitive and behavioral efforts made to manage, tolerate, or reduce external and internal demands and conflicts between them.”* The term “*coping strategies*” refers to cognitive and behavioral attempts to deal with specific external or internal demands (and conflicts between them) that are considered demanding or beyond a person’s resources (Lazarus, 1991, p. 112). An important area for the study of mental health and psychosocial support in conflict-affected communities is the exploration of protective factors, including coping (Tol et al., 2011).

To our knowledge, there is no systematic study examining the coping strategies of refugees and IDPs from the Ukrainian conflict. A variety of methods and paradigms can be used to understand coping (Lazarus and Folkman, 1984; Parker and Endler, 1992). Basic coping strategies in the literature include support seeking, positive cognitive restructuring, problem solving, distraction, and escape-avoidance (Seguin and Roberts, 2015).

Researchers and clinicians could shift the focus from an exclusive emphasis on pathology to the promotion of protective factors by adopting a strategy guided by the concept of resilience. Such a shift in focus would provide the elements necessary to improve each individual’s developmental trajectory, which would later serve as a protective factor in the face of new difficulties.

The aim of this study is therefore to examine the psychological and environmental elements that influence the resilience of Ukrainian refugees and IDPs by analyzing coping strategies and risk and protective factors. Health professionals pursuing a therapeutic strategy to support refugees and IDPs based on the concept of resilience may benefit from identifying protective variables that maintain normative development despite traumatic situations.

## 2. Methods

The quantitative study was analyzed using the Preferred Reporting Items for Systematic Reviews and Meta-Analysis (PRISMA) guidelines (Moher et al., 2009). We outlined our research questions for the 2022–2023 literature search approach, screening phase, and final data extraction in line with our objectives.

First, the inclusion and exclusion criteria were merged with the screening questions (Aromataris and Pearson, 2014). Second, the Population, Intervention, Comparison, Outcome measures, and Study (PICOS) system was used to clarify the questions and establish criteria for the studies (Methley et al., 2014).

The outcomes of interest in this research are coping mechanisms and risk and protective factors used in the context of mental health as defined by World Health Organization (2015) to deal with difficulties arising from armed conflict and forced displacement. The war-affected adult civilian population aged 18 years or older formed the included population. Studies targeting only adolescents and children were not included, as their coping mechanisms are often very different from those of adults.

Studies that focused on internally displaced persons who were forced to leave conflict areas and remain within the borders of their country, as well as refugees who were forced to flee their country due to the conflict, were also included. Studies that focused exclusively on soldiers and war veterans were also not included, as they may have different resources and coping mechanisms than civilians affected by conflict. Both quantitative and qualitative English-language research was included in the review.

### 2.1. Data search, study screening, and analysis

PsycINFO, Pubmed, Scopus, and Science Direct as well as gray literature databases were also searched. The date of first publication was 24 February 2022 (date of the start of the conflict), and the final publication date had no restrictions. A preliminary assessment of relevant publications helped to select the search terms, which included terms for refugees and IDPs as well as typical terms for mental illness. The final keywords were: *“mental health”* AND *“refugee”* AND *“Ukraine”* OR *“IDPs”* on title and abstract for the articles.

English-language or Italian-language empirical research on adult populations was selected. The term “coping” was intentionally not included in the search. Because the concept of coping is so broad, some authors may not have clearly indicated how they would structure their research on coping, but they still included important components of coping that fit the definition and inclusion criteria of this systematic review. The methodological choice to include articles written in English and Italian was dictated by the urgency to have an overview of risk and protective factors and coping strategies to guide potential interventions. However, future works need to consider potential papers written in Ukrainian or Russian.

First, the retrieved papers were downloaded into Mendeley after searching the databases using the search terms. Second, the inclusion/exclusion criteria were checked against the titles and abstracts. Third, the full texts of the included articles were read. Articles that met the above inclusion criteria were included. Fourth, additional relevant publications were found by manually searching the reference lists of the included research papers. Fifth, a thorough analysis of the remaining research papers was conducted.

## 3. Results

**Figure 1:**
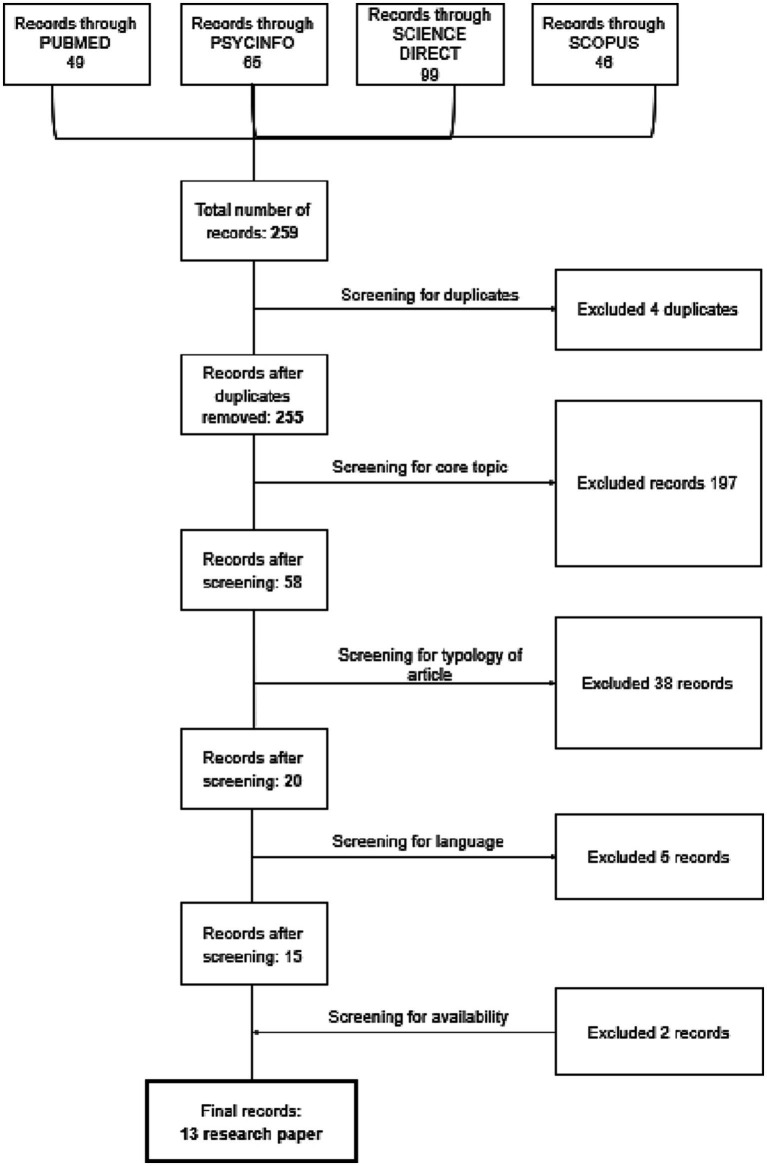
Flow diagram.

Figure 1 shows the results of the five-stage screening process. There were a total of 259 studies (stage 1). After a review of titles and/or abstracts (stage 3), looking for core themes, 197 studies were excluded because they did not deal with coping mechanisms or dealt exclusively with children and adolescents, combatants, and war veterans, populations affected by conflicts that took place more than 10 years before the data collection.

In the next phase, 38 articles were excluded because they were review articles, editorials, or conference summaries. Phase 4 involved a more detailed assessment of the remaining 20 publications. In this phase, five research papers were excluded because they were written in languages other than English. Initially, 13 articles were selected for the final in-depth assessment. All articles were included in the final assessment.

### 3.1. The studies’ characteristics

Thirteen studies were included in this systematic review. Features of the studies are summarized in Table 1.

**Table 1:** Studies’ features.

Study	Study design	Timeframe	Country were the study was conducted	Study population	Sample	Study objectives	Measures	Results
Karatzias et al. (2023)	Cross-sectional study	6 months after Russia’s invasion	Ukraine	Ukranian civilians	2,004 adult parents of children under 18	Assess the type and frequency of exposure to different war-related stressors, the prevalence rates of ICD-11 PTSD and CPTSD, and their relationship in a nationwide sample of parents living in Ukraine during the Russian war.	*War-related stressor:* a list of 34 events was used to assess various stressful experiences people may have had during the war; *The International Trauma Questionnaire (ITQ: 10):* self-report measure to assess PTSD and CPTSD	All participants were exposed to at least one war-related stressor. Mean number of exposures = 9.07 (range = 1–26). 25.9% (95% CI = 23.9, 27.8%) met diagnostic requirements for PTSD and 14.6% (95% CI = 12.9, 16.0%) met requirements for CPTSD. Participants who had the highest exposure to war-related stressors were significantly more likely to meet the requirements for PTSD (OR = 4.20; 95% CI = 2.96–5.95) and CPTSD (OR = 8.12; 95% CI = 5.11–12.91) compared to the least exposed
Khraban (2022)	Qualitative study	first 15 days of war (24.02.2022 to 10.03.2022)	Northern Ukraine	Ukranian civilians	Ukrainian sector of the social network Facebook on the pages of the groups “Boyarka and community news” (23.4 thousand participants) and “Boyarka. Boyarka community” (15.0 thousand participants). A total of 582 posts and comments to them were analyzed.	Identify topical coping strategies that were used by civilians of northern Ukraine during the first 15 days in the zone of military conflict.	Posts and comments to them, which contain linguistic markers of coping behavior (coping) and which have been posted during the period from 24.02.2022 to 10.03.2022 in the Ukrainian sector of the social network Facebook on the pages of the groups “Boyarka and community news.”	During the first five days non-adaptive emotionally focused coping strategies become in high demand. On days 6–10 of military conflict the civilians turn to problem-oriented, collective in nature coping strategies. On days 11–15 after the beginning of military conflict coping strategies aimed at creating a positive emotional state become predominant
Chudzicka-Czupała et al. (2023a)	Cross-sectional study	first stages of war (8 March and 26 April 2022)	Ukraine, Poland, Taiwan	Ukranian, Polish, and Taiwanese civilians	1,598 participants (362 from Ukraine, 1,051 from Poland, and 185 from Taiwan)	Compare psychological distress and coping strategies among people living in Ukraine, Poland, and Taiwan and examine whether the associations between various coping strategies and psychological distress differed among people of various countries during the initial stage of the 2022 War in Ukraine.	*Hopelessness about the ongoing war:* one item (‘I feel hopeless about the current war in Ukraine’) to assess the current level of hopelessness of the respondents toward the 2022 War in Ukraine; *Brief Coping Orientation to Problems Experienced Inventory:* to measure how our participants behaved in response to the recent events related to the war; *21-Item Depression, Anxiety and Stress Scale:* to measure the three dimensions of emotional state in the preceding week; *Impact of Event Scale–Revised:* to measure the three dimensions of the psychological stress reactions (i.e., intrusion, avoidance, and hyperarousal) of the respondents to the war.	Psychological distress and adoption of coping strategies differed across people of various countries. Among Taiwanese and Polish respondents, avoidant coping strategies were most strongly associated with all categories of psychological distress compared with problem- and emotion-focused coping strategies. However, the associations of various coping strategies with psychological distress differed to a less extent among Ukrainian respondents. In addition, problem- and emotion-focused coping strategies had comparable associations with psychological distress among the people of Ukraine, Poland, and Taiwan.
Oviedo et al. (2022)	Qualitative study	Started in February 2022. Refugees were interviewed between 1 and 3 months since they left Ukraine	Poland, Italy, and Spain	Ukranian refugees	94 Ukranian refugees	Identify: (1) what are the main stressors that are afflicting Ukrainian refugees; (2) what are the main coping strategies that they practice and advise for others; (3) what is the image they have of the hosts and other people they have met; (4) what are their current state and expectations; and finally, (5) what role does religion and religious prayer play in their stressing context	semi-structured interviews	The data obtained pointed to a plurality of coping and resilience strategies. Maintaining communication with separated loved ones as well as experiencing accompaniment by helpers and hosts emerged as principal elements for coping and resilience. It was found that a prior development of interior life or practice of prayer served as psychological “capital” that increased their resilience.
Chudzicka-Czupała et al. (2023b)	Cross-sectional study	First stages of war (8 March and 26 April 2022)	Ukraine, Poland, and Taiwan	Ukranian, Polish, and Taiwanese civilians	1,626 participants (Poland: 1053; Ukraine: 385; Taiwan: 188)	Assess and compare mental health status, coping strategies, and views on the Russo-Ukrainian war in populations from Poland, Ukraine, and Taiwan, as well as identify demographic socio-and economic factors associated with depression, anxiety, stress, and post-traumatic stress levels	*Brief Coping Orientation to Problems Experienced Inventory*: to measure how our participants behaved in response to the recent events related to the war; *21-Item Depression, Anxiety and Stress Scale*: to measure the three dimensions of emotional state in the preceding week; *Impact of Event Scale–Revised:* to measure the three dimensions of the psychological stress reactions (i.e.intrusion, avoidance, and hyperarousal) of the respondents to the war	Ukrainian participants reported signifcantly higher DASS-21 (p < 0.001) and IES-R (*p* < 0.01) scores than Poles and Taiwanese. Taiwanese reported signifcantly higher avoidance scores (1.60 ± 0.47) than the Polish (0.87 ± 0.53) and Ukrainian (0.91 ± 0.5) participants (*p* < 0.001). More than half of the Taiwanese (54.3%) and Polish (80.3%) participants were distressed by the war scenes in the media. More than half (52.5%) of the Ukrainian participants would not seek psychological help despite a significantly higher prevalence of psychological distress. Multivariate linear regression analyses found that female gender, Ukrainian and Polish citizenship, household size, self-rating health status, past psychiatric history, and avoidance coping were significantly associated with higher DASS-21 and IES-R scores after adjustment of other variables (*p* < 0.05)
Xu et al. (2023)	Cross-sectional study	Initial period of the Russian invasion (March 19–31, 2022)	Ukraine	Ukranian civilians	801 ukranian civilians	Provide the prevalence rates of symptoms of psychological distress, anxiety, depression, and insomnia; and to link them with Ukrainians’ productive coping strategies during the war	*Kessler Psychological Distress scale (K6):* to assess psychological distress; *Generalized Anxiety Disorder-2:* to assess anxiety: *Patient Health Questionnaire-2*: to assess depression; *Insomnia Severity Index-4*: to assess insomnia; *Brief COPE:* to assess models of coping	Of 801 Ukrainian adults, 52.7% had symptoms of psychological distress (mean = 13.3 [SD = 4.9]); 54.1% of them reported symptoms of anxiety (mean = 2.9 [SD = 1.7]); 46.8% reported symptoms of depression (mean = 2.6 [SD = 1.6]). Symptom criteria for insomnia were met by 97 respondents (12.1%) (mean = 10.4 [SD = 4.2]). Demographic variables (including gender, living in an urban area, having children or elderly persons in the household, living in an area occupied by Russian forces) were associated with symptoms of distress, anxiety, depression, and insomnia. The productive coping strategies of using instrumental support, behavioral disengagement, self-distraction, and planning were significantly associated with mental health symptoms
Rizzi et al. (2022)	Cross-sectional study	Between March and June 2022	Ukraine and Poland	Ukranian refugees and IDPs	352 Ukranian refugees and 271 Ukranian IDPs	Explore the mental health and well-being of Ukranian refugees and IDPs who experienced transit conditions; examine the association of mental health and well-being with refugees’ and IDPs’transit conditions and explore the protective role of family in building resilience by being open and (re)examining the idea of family	*A revised version of the DSM 5 TR Rated Level 1 Cross Cutting Symptoms:* to assess menatl health. IDPs and refugees were asked to indicate the extent of their depression, anger, anxiety, and sleep disturbances on a Likert scale of 0–4 [none (0), mild (1), moderate (2), severe (3), or very severe (4)]. Two open-ended questions: what helps you feel better during this traumatic time and what worries you the most? to deepen the understanding of family resilience	Most of the samples (refugees and IDPs) reported high or very high levels of anxiety, depression, and sleep disturbances. Moreover, results highlighted how being close to families or being able to keep in touch with them work as a protective factor in enhancing resilience, as well as a support network
Miliutina et al. (2023)	Cross-sectional study	Between February and April 2022	Ukraine	Ukranian civilians	115 ukranian families (45 in Kyiv, 12 Internally displaced, and 58 in evacuation in Europe countries)	Examine the influence of pets and relationships with them on maintaining the emotional state and the decision-making process of the Ukrainian residents during the war	In-depth interviews; the multidimensional psychological well-being model proposed by Ryff (the 42-item Psychological Wellbeing).	15% of adults experienced an improvement in their emotional state (residents who remained in Kyiv), 23% had a sense of shame (associated with inconvenience, that animals cause to other people), 42% have a feeling of anxiety (associated with concern for the life and health of an animal), 20% have a feeling of guilt in relation to abandoned and (or) dead animals. The migrants and refugees who took their pets with them showed an increase in the level of subjective well-being
Kokun (2022)	Exploratory cross-sectional study	Between June and July 2022	Ukraine	Ukranian civilians	1,243 ukranian civilians	Determine the possible differences in indicators of psychological and physical health in the Ukrainian population depending on the degree of employment, financial, and housing losses caused by the war	Ukranian adapted versions of: *the Short Screening Scale for DSM–IV PTSD:* to assess PTSD; *the Connor-Davidson Resilience Scale, 10-Item Version:* to assess resilience; *the General Self-Efficacy Scale:* to evaluate individuals’ perception of their competence in effectively manage stressful situations; *the Giessen Subjective Complaints List:* to quantify physical complaints	The sample showed significantly high levels of PTSD symptoms and physical complaints. Helplessness in the population caused by the war can be considered as an important mediating factor deteriorating the Ukrainian population’s psychological and physical health as a result of employment and, especially, financial and housing losses. Secondly, such war-induced helplessness naturally reduces a person’s ability to effectively search for work and earn money in new, unfavorable conditions, as well as his/her ability to minimize possible financial losses
Talabi et al. (2022)	Descriptive survey research design	Not specified	Nigeria	Ukranian refugees	476 Nigerian refugees	Determine the utilization of social media storytelling for seeking and receiving help among Nigerian refugees who were displaced by the war between Russia and Ukraine	An online questionnaire was used to collect data for this study. The questionnaire sought both demographic and psychographic data. The response format was a five-point Likert scale that ranges from strongly agree to strongly disagree	The result of the study showed a significant positive correlation between social media storytelling and help-seeking and help-receiving among Nigerian refugees who were displaced by the war. The additional result showed that communication systems determined the promptness in receiving help. In particular, the macro-level was found to be more effective in promptness of help received than meso and micro levels
Kurapov et al., 2023	Cross-sectional study	From May 5 to May 17, 2022	Ukraine	Ukranian university students and personnel	589 university students (69.2%) and faculty/staff (personnel; 30.8%)	Investigate the impact of the war on the mental and emotional well-being of Ukrainian civilians-university students and personnel	*Modified version (FWS-Ukraine) of the Fear of War Scale:* to assess fear of war; *the Brief Resilience Scale*: to assess resilience; *the Short Burnout Measure:* to assess burnout; *the De Jong Gierveld 6-Item Loneliness Scale*	Most respondents (97.8%) reported deterioration of their psycho-emotional status including depression (84.3%), exhaustion (86.7%), loneliness (51.8%), nervousness (84.4%), and anger (76.9%)—students more than personnel, females more than males. The use of substances (i.e., tobacco, alcohol, pain relievers, and sedatives) has increased as well as loneliness associated with fear, burnout and lower resilience. However, despite these conditions, 12.7% of the respondents have reported the war has not affected them
Hamama-Raz et al. (2022)	Cross-sectional study	From April 7 to April 15, 2022	Ukraine	Ukranian civilians	2,000 Ukranian civilians	Explore predictors of patriotism attitudes among Ukrainians during wartime; and examine associations between patriotic attitudes and PTSD symptoms among Ukrainians during the conflict, alongside socio-demographic and war-related variables	Demographic information (sex, age, marital status, and having children under the age of 16 and region), with war related information assessed by three questions: “Do you have relatives that were wounded during the current war?”; “Do you have relatives that were killed in the current war?”; and “Do you have relatives that left Ukraine because of the current war?” (All rated Yes/No). Patriotic attitudes were measured with 20 items on a scale from 1 = Not true for me to 4 = Very true for me. (e.g., “I feel a sense of belonging to Ukraine no matter the challenges”; “It is necessary for me to serve my country”). the *International Trauma Questionnaire (ITQ):* to measure PTSD symptoms	Hierarchical regressions found that having relatives that were wounded or that left Ukraine because of the war and those coming from a Ukrainian speaking region were associated with patriotic attitudes. Patriotic attitudes were positively associated with elevated risk for PTSD symptoms
Długosz (2023)	Cross-sectional study	Between April 15, 2022 and May 10, 2022	Poland	Ukranian refugees	737 ukranian refugees in Poland	Diagnose mental health disorders with the use of the RHS-15 scale. Another aim of the study is the observation of strategies for coping with stress.	*The Refugee Health Screener-15 (RHS-15):* to assess mental distress	The analysis of responses on the RHS-15 scale has shown that depression, anxiety disorders and PTSD were observed among 73% of respondents, whereas 66% of the respondents display psychological distress. The analyses have shown that higher levels of mental health disorders were observed among women and refugees who do not speak Polish. Younger respondents experienced a higher psychological distress. The results of the study also indicate that the refugees more often implemented problem-focused strategies. The analysis has shown that the respondents who followed active strategies scored the lowest on RHS-15. The emotion-focused strategies, such as praying, diverting attention by becoming involved in different activities or taking sedatives were not effective. The highest levels of disorders were present among the refugees who indicated resignation

Most of the studies (*n* = 10) were cross-sectional studies (Hamama-Raz et al., 2022; Kokun, 2022; Rizzi et al., 2022; Długosz, 2023; Karatzias et al., 2023; Kurapov et al., 2023; Miliutina et al., 2023; Xu et al., 2023; Chudzicka-Czupała et al., 2023a,b), two of them were qualitative studies (Khraban, 2022; Oviedo et al., 2022) and one had a descriptive survey research design (Talabi et al., 2022). Four of the included studies (Khraban, 2022; Xu et al., 2023; Chudzicka-Czupała et al., 2023a,b) investigated the first stages of the war, most of them (*n* = 7) were conducted between 1 and 5 months after Russian invasion (Hamama-Raz et al., 2022; Kokun, 2022; Oviedo et al., 2022; Rizzi et al., 2022; Długosz, 2023; Kurapov et al., 2023; Miliutina et al., 2023), only one study (Karatzias et al., 2023) was conducted 6 months after Russia’s invasion and in one study (Talabi et al., 2022), the timeframe was not specified. Six countries were represented: Ukraine (*n* = 10; Hamama-Raz et al., 2022; Khraban, 2022; Kokun, 2022; Rizzi et al., 2022; Karatzias et al., 2023; Kurapov et al., 2023; Miliutina et al., 2023; Xu et al., 2023; Chudzicka-Czupała et al., 2023a,b), Poland (*n* = 5; Oviedo et al., 2022; Rizzi et al., 2022; Długosz, 2023; Chudzicka-Czupała et al., 2023a,b), Taiwan (*n* = 2; Chudzicka-Czupała et al., 2023a,b), Italy (*n* = 1; Oviedo et al., 2022), Spain (*n* = 1; Oviedo et al., 2022), and Nigeria (*n* = 1; Talabi et al., 2022). The majority of the studies (*n* = 10) included in their sample Ukrainian IDPs (Hamama-Raz et al., 2022; Khraban, 2022; Kokun, 2022; Rizzi et al., 2022; Karatzias et al., 2023; Kurapov et al., 2023; Miliutina et al., 2023; Xu et al., 2023; Chudzicka-Czupała et al., 2023a,b) while four studies included in their sample Ukrainian refugees (Oviedo et al., 2022; Rizzi et al., 2022; Talabi et al., 2022; Długosz, 2023). Among the studies examined, eight studies (Khraban, 2022; Oviedo et al., 2022; Rizzi et al., 2022; Długosz, 2023; Karatzias et al., 2023; Xu et al., 2023; Chudzicka-Czupała et al., 2023a,b) specifically included among their objectives that of investigating risk and/or protective factors and/or coping strategies. Among the instruments used to assess risk and/or protective factors there were: the War-related stressor (Karatzias et al., 2023), a list of 34 events was used to assess various stressful experiences people may have had during the war, interviews (Oviedo et al., 2022; Miliutina et al., 2023), two open-ended questions: “what helps you feel better during this traumatic time and what worries you the most?” (Rizzi et al., 2022), and the De Jong Gierveld 6-Item Loneliness Scale (Kurapov et al., 2023). The coping strategies were assessed using linguistic markers of coping behavior in posts and comments on social networks (Khraban, 2022), the Brief Coping Orientation to Problems Experienced Inventory (Xu et al., 2023; Chudzicka-Czupała et al., 2023a,b), and interviews (Oviedo et al., 2022; Miliutina et al., 2023); Two open-ended questions: “what helps you feel better during this traumatic time and what worries you the most?” (Rizzi et al., 2022), the Connor-Davidson Resilience Scale, 10-Item Version (Kokun, 2022), the General Self-Efficacy Scale (Kokun, 2022), and the Brief Resilience Scale (Kurapov et al., 2023).

### 3.2. Risk and protective factors

Table 2 shows risk and protective factors included in the studies examined.

**Table 2:** Risk and protective factors.

Study	Risk factors	Protective factors
Karatzias et al. (2023)	Air raid sirens (99.0%), experiencing extreme financial hardship (74.6%), having to take shelter in an underground location (72.5%), seeing/hearing bombing and artillery fire (67.3%), and witnessing the destruction of local infrastructure (64.1%)	/
	Cultural difference in gender role	
	Financial security (e.g., experienced extreme financial hardship), and damage to their local environment (e.g., destruction of local infrastructure)	
	Mental health disorder	
	Lack of sleep	
	Relatives wounded	
	Loved ones displaced	
	Lack of essential care	
	Someone close was kidnapped	
	Move to another country	
	Torture/kidnapping	
	Experience sexual violence	
Khraban (2022)	Air raid sirens, alrert music from loudspeakers, lack of primary needs (water and food), and lack of normality	Pets support
		Faith
		Strong sense of ethnic and national identity
		Communicational support
		Neighbor support
		Humor
		Sense of the belonging to the nation
		Self-compassion
		Provision of physical safety and supportive staff in refugee camps
Chudzicka-Czupała et al. (2023a)	Exposure to war information	/
	Mental health disorder	
Oviedo et al. (2022)	Shelter, leave home, and unfamiliar environment	Just 11.7% were being assisted by friends or relatives, although over 77% expected to find a better place to stay than the places they left behind, places often characterized by bombing and other great threats
		Children
	Being sometimes close to bombing zones or rail stations under attack for several days; not being able to sleep; being under very cold temperatures; and experiencing scarcity of food	Kindness and appreciation
		Gratitude and compassion
	Parental mental health	Faith to get back to Ukraine
		Religion
Chudzicka-Czupała et al. (2023b)	Mental health disorders	/
	Female gender	
	Self-rated health status	
Xu et al. (2023)	/	/
Hamama-Raz et al. (2022)	Wound relatives	/
	Mental health disorders	
	Relative left in Ukraine	
	Perceived threat	
	Patriotism	
Rizzi et al. (2022)	Duration of the trip	Have a support network
		Religion
		Planning the future
	Being IDPs	Feeling safe
		Be able to engage in leisure activities
	Lack of contact with relatives	Help other people
		Talk about the war and give an explanation about it
Miliutina et al. (2023)	Difficulties with pets’ transportation prevent timely evacuation from dangerous areas	Pets helped maintain the normative emotional state of children
Kokun (2022)	Helplessness and hopelessness	/
Talabi et al. (2022)	/	Use of social media story telling in seeking support
Kurapov et al. (2023)	Use of substances (i.e., tobacco, alcohol, pain relievers, and sedatives)	/
	Student status	
	Female gender	
Długosz (2023)	Female gender	/
	Resignation	

**Figure 2:**
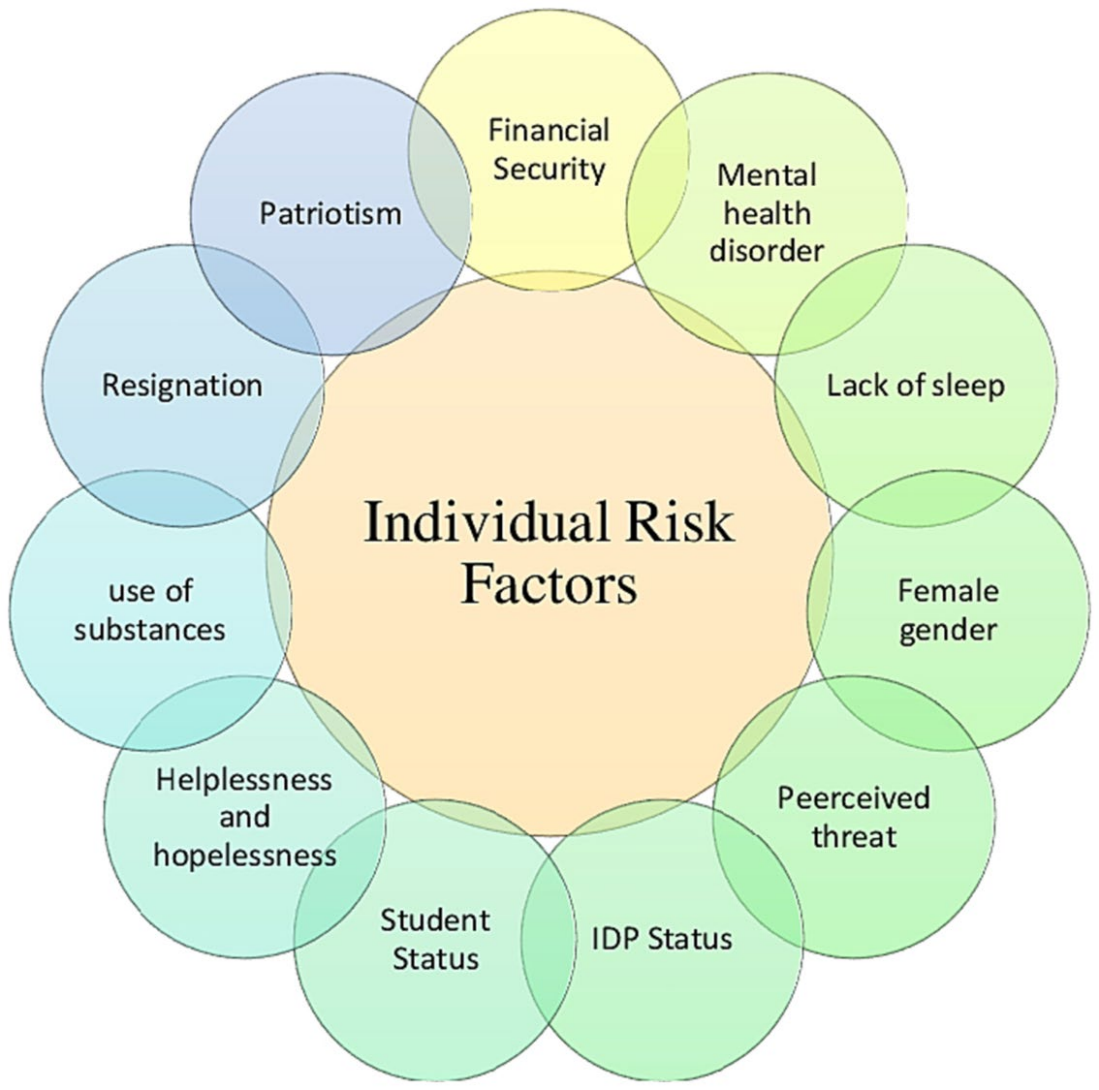
Individual risk factors associated with Ukranian refugees and lDPs resilience.

Specifically, with respect of risk factors, individual risk factors are summarized in Figure 2.

**Figure 3:**
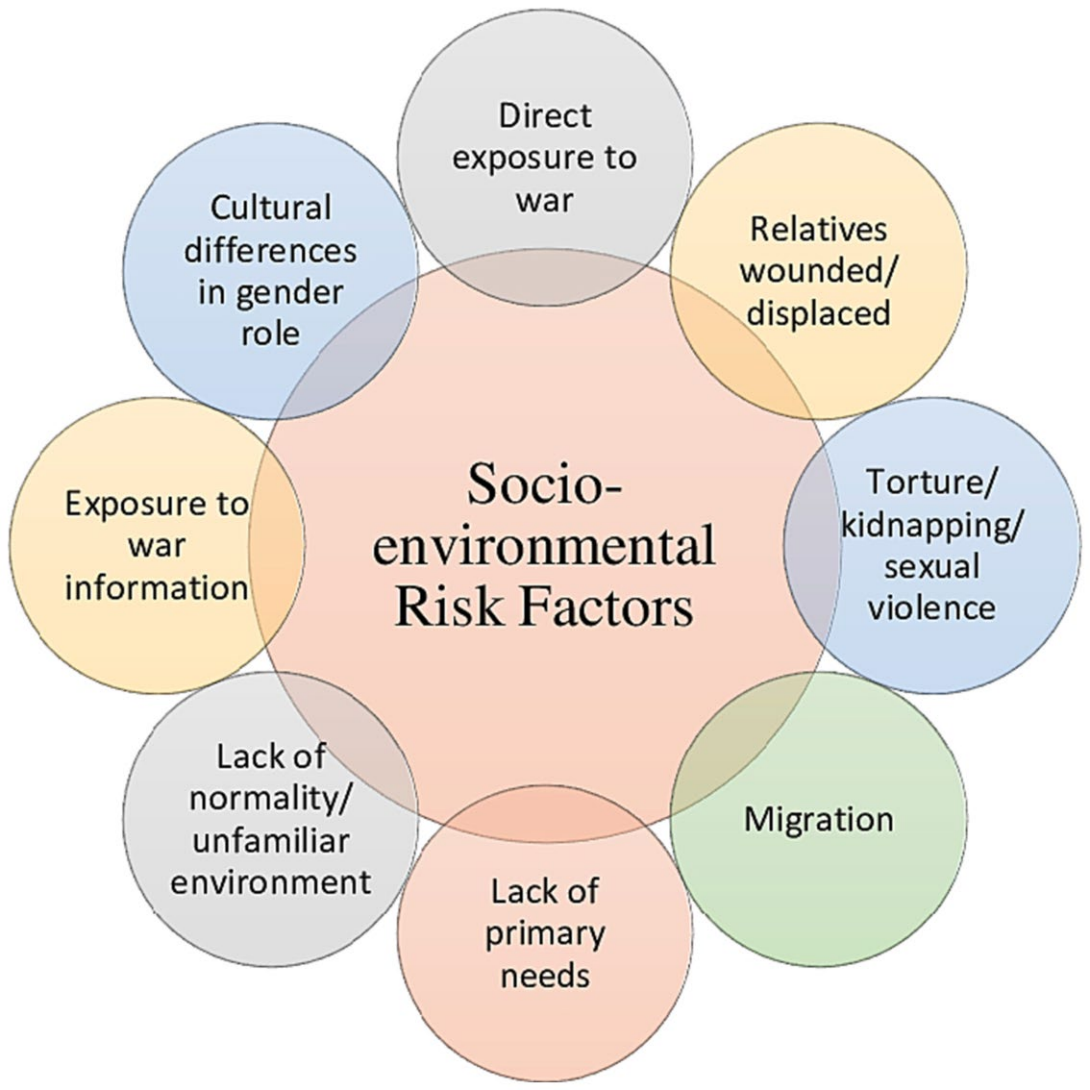
Socio-environmental risk factors associated with Ukranian refugees and IDP’s resilience.

Figure 3 summarizes the main socio-environmental risk factors.

From the studies reviewed, the individual risk factors that stand out the most were mental health disorders (Hamama-Raz et al., 2022; Chudzicka-Czupała et al., 2023a,b), female gender (Długosz, 2023; Kurapov et al., 2023; Chudzicka-Czupała et al., 2023b) and lack of sleep (Oviedo et al., 2022; Karatzias et al., 2023). On the other hand, the most frequently reported socio-environmental risk factors were direct exposure to war (Khraban, 2022; Oviedo et al., 2022; Karatzias et al., 2023), relatives wounded/displaced (Hamama-Raz et al., 2022; Rizzi et al., 2022; Karatzias et al., 2023), migration (Oviedo et al., 2022; Rizzi et al., 2022; Karatzias et al., 2023), lack of primary needs (Khraban, 2022; Oviedo et al., 2022; Karatzias et al., 2023), and lack of normality/unfamiliar environment (Khraban, 2022; Oviedo et al., 2022; Karatzias et al., 2023).

**Figure 4:**
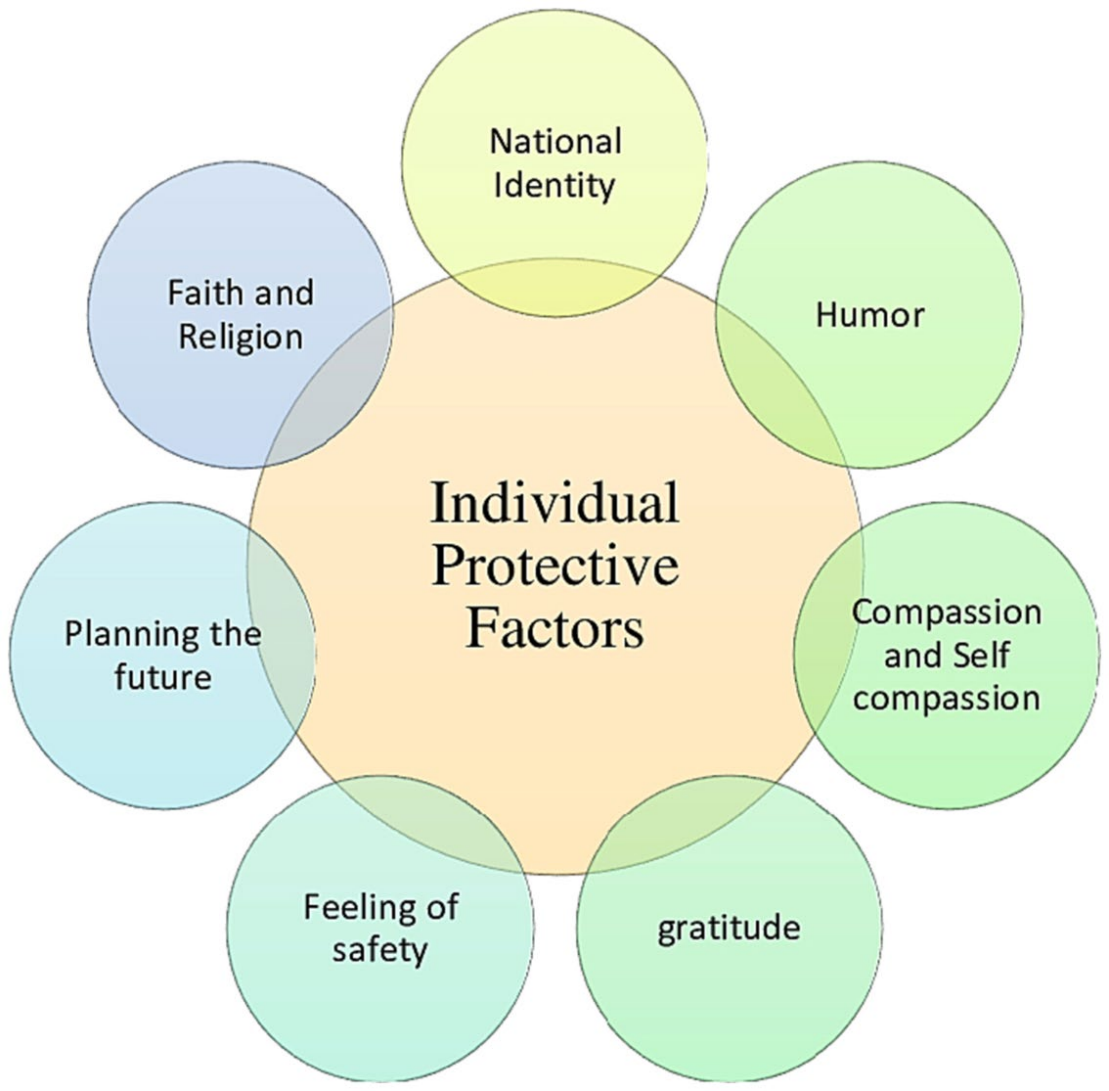
Individual protective factors associated with Ukranian refugees and lDPs resilience.

**Figure 5:**
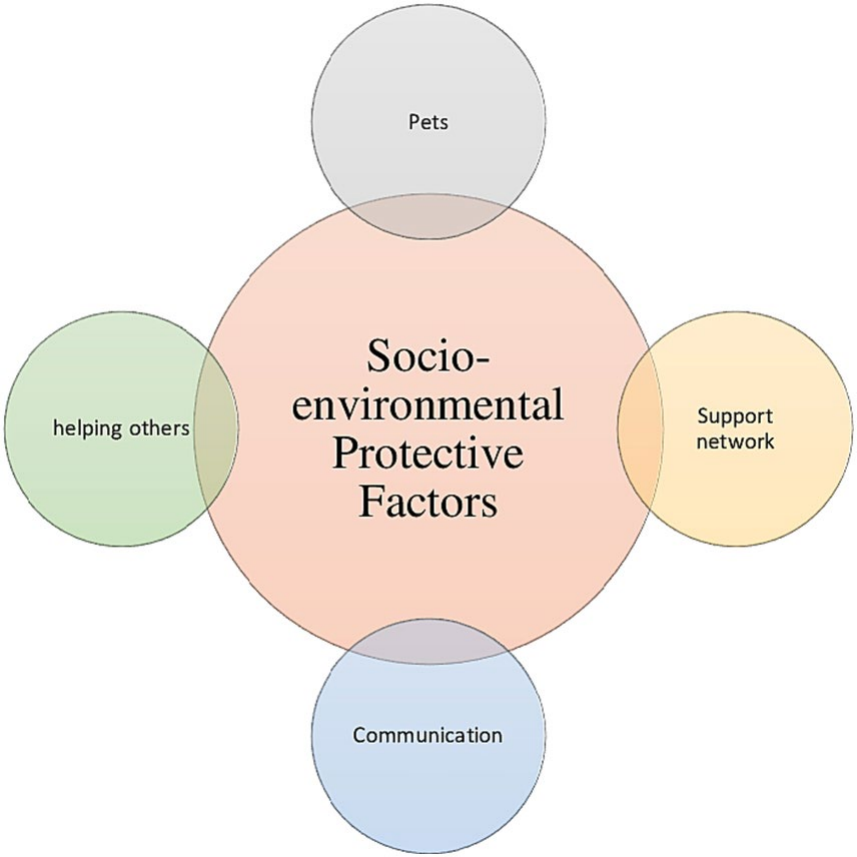
Socio-environmental protective factors associated with Ukranian refugees and lDPs resilience.

With respect of protective factors, Figure 4 summarizes the main individual ones, while the main socio-environmental protective factors are summarized in Figure 5.

Faith and religion were the most reported individual protective factors (Khraban, 2022; Oviedo et al., 2022; Rizzi et al., 2022), followed by the feeling of safety (Khraban, 2022; Rizzi et al., 2022) and self-compassion/compassion (Khraban, 2022; Oviedo et al., 2022). Considering socio-environmental factors, the most cited one was the presence of a support network (Khraban, 2022; Oviedo et al., 2022; Rizzi et al., 2022; Talabi et al., 2022), that included friends and relatives as well as stuff in refugee camps, followed by communication (Rizzi et al., 2022; Talabi et al., 2022) and the presence of pets (Khraban, 2022; Miliutina et al., 2023).

### 3.3. Coping strategies

Table 3 summarizes the coping strategies reported in the studies examined.

**Table 3:** Coping strategies.

	Coping strategies							
Study	Emotion-focused		Problem-focused		Avoidance		Faith-based	Sense of belonging
	Support (seeking)	Others	Positive cognitive restructuring	Problem solving	Distraction	Escape-avoidance		
Khraban (2022)	From the sixth day onward: pursuit of altruism, emotional and instrumental support provision	In the first 5 days: Emotional ventilation, blaming others, and catastrophizing; From the sixth day onward: emotional self-regulation	In the first 5 days: Challenging the negative beliefs and thoughts of others; From the sixth day onward: positive revaluation; and days 11–15 after the beginning of hostilities: humor	From the sixth day onward: active coping	Days 11–15 after the beginning of hostilities: indulging in the desires, distraction activities	/	In the first 5 days and days 11–15 after the beginning of hostilities: hope and focus on the future	From the sixth day onward: pursuit of unity; days 11–15 after the beginning of hostilities: creating a sense of belonging
Chudzicka-Czupała et al. (2023a)	Adopted mostly by Taiwanese respondents; adopted more by Polish respondents (compared to the Ukranian respondents)		Adopted mostly by Taiwanese respondents; adopted more by Ukranian respondents (compared to polish respondents)		Adopted mostly by Taiwanese respondents (no difference between the Polish and Ukranian respondents)		/	/
Oviedo et al., 2022	Relationships (including family, friends and others)	Therapy; positive experiences; feeling safe	Positive thinking	/	Activity (working, sport)	/	Interior life (including prayers, belief, confidence; hope); good expectations (hope; victory in the war)	/
Rizzi et al. (2022)	Being close to the family, support network (family, friends, and volounteers); helping/caring	Feeling safe	Meaning-making communication	/	Engaging in leisure activities	/	Religion and hope for the future	/
Hamama-Raz et al. (2022)	/	/	/	/	/	/	/	Patriotism
Chudzicka-Czupała et al. (2023b)	Adopted mostly by Taiwanese respondents; adopted more by Polish respondents (compared to the Ukranian respondents)		Adopted mostly by Taiwanese respondents; adopted more by Ukranian respondents (compared to polish respondents)		Adopted mostly by Taiwanese respondents (no difference between the Polish and Ukranian respondents)		/	/
Xu et al. (2023)	Seeking instrumental support (negatively associated with psychological distress but positively associated with anxiety)	/	Positive reframing (not related to Ukranians’ mental health symptoms); humor (not related to Ukranians’ psychological symptoms)	Active coping (positively associated with anxiety) and planning (positively associated with insomnia)	Self-distraction (positively associated with symptoms of psychological distress but negatively associated with anxiety, depression, and insomnia) Behavioral disengagement (negatively associated with psychological distress)	/	Religion (not related to Ukranians’ psychological symptoms)	/
Miliutina et al. (2023)	/	/	/	Presence of pets reinforced ukranian families decision-making process	/	/	/	/
Kokun (2022)	/	/	/	Self-efficacy	/	/	/	/
Talabi et al. (2022)	Use of social media storytelling	/	/	/	/	/	/	/
Kurapov et al. (2023)	/	/	/	/	/	Use of substances (i.e., tobacco, alcohol, pain relievers, and sedatives)	/	/
Karatzias et al. (2023)	/	/	/	/	/	/	/	/
Długosz (2023)	/	/	/	Those participants scored less at the RHS-15	Diverting attention by becoming involved in different activities (was found not to be an effective strategy)	Taking sedatives (was found not to be an effective strategy)	Praying (was found not to be an effective strategy)	/

Five main categories were identified: emotion-focused strategies, problem-focused strategies, avoidance, faith-based strategies, and the ones based on sense of belonging. The four studies that investigated the first stages of war identified the use of a variety of coping strategies among Ukrainian civilians. Khraban (2022) highlighted how, during the first 5 days, non-adaptive emotionally-focused coping strategies have been the most widely adopted, while on days 6–10 of military conflict the civilians turn to problem-oriented coping strategies. On days 11–15 after the beginning of military conflict, coping strategies aimed at creating a sense of belonging and based on a sense of hope and faith for the future become predominant. Similarly, Xu et al. (2023) emphasized a massive use of emotion-, problem focused strategies and avoidance. Moreover, they found that the productive coping strategies of using instrumental support, behavioral disengagement, self-distraction, and planning were significantly associated with mental health symptoms. Apparently, faith-based strategies were found not to be related to Ukrainian civilians’ psychological symptoms. Furthermore, the works of Chudzicka-Czupała et al. (2023a,b) offer a cross-cultural comparison that highlighted how psychological distress and adoption of coping strategies differ across people of various countries. The associations of various coping strategies with psychological distress differed to a less extent among Ukrainian respondents compared to Taiwanese and Polish ones. However, problem- and emotion-focused coping strategies had comparable associations with psychological distress among the three samples. As the war progressed, Oviedo et al. (2022) found that, for refugees, strategies aimed at maintaining a solid social network and communication with loved ones were the most effective strategies. Faith-based strategies, in that stage of the war, was found to serve as psychological “capital” that increased their resilience.

Similarly, Rizzi et al. (2022) have identified how being close to families or being able to keep in touch with them, as well as having a support network worked as a protective factor in enhancing resilience. Even in this research, religion and hope for the future were found to be effective strategies. The work of Talabi et al. (2022) specifically stressed how the use of social media storytelling was an effective strategy in help-seeking and help-receiving among refugees. Długosz (2023) found that the refugees more often implemented problem-focused strategies. The respondents who followed active strategies scored the lowest on RHS-15. On the other hand, the strategies identified by the author as emotion-focused ones, such as praying, diverting attention by becoming involved in different activities or taking sedatives were not effective. The highest levels of disorders were present among the refugees who indicated resignation. Miliutina et al. (2023) focused their work on pets and found that, in terms of problem-solving strategies, difficulties with pets’ transportation prevent timely evacuation from dangerous areas, however, migrants and refugees who took their pets with them showed an increase in the level of subjective well-being. Among civilians, Kurapov et al. (2023) studied the effects of war in a population of Ukrainian university students and personnel and highlighted a massive use of avoidance that was found not to serve as an effective strategy in enhancing their resilience. Furthermore, Hamama-Raz et al. (2022) have focused their research on the study of patriotic attitudes that were found to be associated with having relatives that were wounded or that left Ukraine because of the war and coming from a Ukrainian speaking region. Patriotism was also found to be positively related to elevated risk for PTSD symptoms. Lastly, Kokun (2022) pointed out in his work how the progression of war and what this entails naturally diminishes problem-solving skills and therefore, civilians with a greater sense of self-efficacy are those who are more resilient.

## 4. Discussion

This study systematically reviewed the current global literature on the psychological and environmental elements that influence the resilience of Ukrainian refugees and IDPs by analyzing coping strategies and risk and protective factors. Studies identified by the search varied with regard to context, samples, study design, timeframe, measurements, and approach to data analysis. War trauma can elicit various physical and psychological responses as natural reactions to overwhelming and prolonged stress. However, these reactions can be distressing for individuals and their families. Moreover, it is important to recognize that the impact of war trauma and associated stressors persists even after individuals have left the war zone, as the challenges of resettling in a new host country and finding a new home can intensify these concerns. Following experiences like war, individuals exhibit diverse reactions based on their personal attributes and the nature of the stressful events. Certain personal factors can heighten the risk of more pronounced or prolonged psychological and psychosocial challenges following wartrauma. These factors encompass, for example, being female, facing poverty and socioeconomic hardships, experiencing pre-existing or ongoing psychological issues or encountering family dysfunction (Opaas, 2022). Furthermore, as emerged in this review, the individual risk factors are related to feelings of uncertainty and insecurity due to an unresolved legal status. Consequently, refugees and IDPs often live in fear, poverty, and isolation, separated from their families (Hoffman, 2011). Moreover, they frequently express a sense of hopelessness and lack of control over their circumstances and over their future (Hoffman, 2011; Bjertrup et al., 2018). The absence of employment opportunities yields not just economic ramifications but also psychological effects since it deprives individuals of routine, stability, social connections, and a sense of safety and security. Thus, the waiting process exacerbates their psychological decline by fostering uncertainty, insecurity, isolation, and a sense of hopelessness, thereby stalling their lives (Hoffman, 2011). Considering that unresolved status, in line with our findings, Seguin and Roberts (2015) in their review present evidence of a stronger association of poor mental health with being internally displaced compared to being a refugee, as revealed in the systematic review by Porter and Haslam (2005). Beside these factors, it is crucial to remember that, during times of war, mass conflicts, or instances of individual violence, the distress and anguish experienced by individuals can be traced back to the deliberate actions and choices of fellow human beings. These acts are typically intentional and purposeful in nature. Some examples of intentional acts of violence include armed assaults, torture, sexual assault, domestic violence, and child abuse. Extensive research has demonstrated that individuals who endure violence intentionally inflicted by others tend to exhibit the most severe reactions to such experiences (Briere and Scott, 2014). Indeed, exposure to violence has been established as the most substantiated factor contributing to the risk of subsequent psychological disturbances. The level of direct exposure to threat (Goldstein et al., 1997; Allwood et al., 2002; Morgos et al., 2007), the cumulative number of adverse events (Mollica et al., 1997; Thabet et al., 2004; Morgos et al., 2007; Mels et al., 2010), and the duration of exposure (Ahmad et al., 2000) consistently heighten the likelihood of experiencing mental health symptoms. It is important to note that risks are not solely increased by actual or threatened violence toward an individual but also by witnessing violence inflicted upon others (Goldstein et al., 1997; Ahmad et al., 2000). The nature of the event itself also plays a significant role, with those events that directly endanger or disrupt the individual, family, or home having particularly profound consequences. Specific events such as house searches (McCallin, 1988), witnessing the death, injury, or torture of a family member (Goldstein et al., 1997), abduction, hiding for protection, rape, being coerced into harming relatives (Morgos et al., 2007), and the duration of captivity (Ahmad et al., 2000) are all factors associated with an elevated risk of psychological difficulties. Furthermore, as evidenced by this review, sustained physical distress resulting from sirens and bombings contributes to emotional distress and trauma, ultimately impacting physical health and overall well-being. Following exposure to highly threatening circumstances, individuals may experience immediate physical reactions such as sleep disturbances, nightmares, somatization (Balaban et al., 2005), restlessness, hyperarousal, heightened vigilance, feelings of weakness, and physical sensations of numbness (World Health Organization, 2015). Refugees and IDPs often encounter more intense traumatic experiences. This is not only due to the circumstances that forced them to flee but also because of the physical stressors endured in migrant and refugee camps, as well as during the resettlement process. Thus, they exhibit elevated rates of stress, depression, post-traumatic stress disorder (PTSD), and various other physical and psychiatric issues. The aftermath of war-related disasters often brings about broken homes, disrupted infrastructures, and the displacement of individuals, leading to a loss of their sense of place and a disruption of their identity (Anjum et al., 2023). Being compelled to reside in an unfamiliar environment could amplify these difficulties. Moreover, the increase in the number of refugees within the host community may result in a lack of hospitality, leading to additional exclusion and marginalization of the refugee population. Additionally, other risk factors that contribute to the decline in psychological well-being during the asylum-seeking process include the inability to meet social roles and expectations (Simich et al., 2010). Consequently, the asylum-seeking process, combined with the uncertainty of living in a state of limbo, could impede social integration within the host community. Given all of this, when it comes to protective factors and coping strategies, the use of faith, spirituality, and religious practices by refugees and internally displaced persons to navigate their circumstances is comprehensible (Kramer and Bala, 2004; Posselt et al., 2018). These practices provide a sense of normalcy and opportunities for social connections. Indeed, our findings highlighted that the strategies aimed at creating a sense of belonging, fostering hope, and maintaining faith in the future were identified as effective coping mechanisms that helped increasing refugees and IDPs’ resilience (Oviedo et al., 2022; Rizzi et al., 2022). Moreover, they employ various behavioral strategies, such as engaging in physical activities, pursuing hobbies, and watching movies, as means of distraction from their current situation. In this review, avoidance strategies were frequently observed, however, in most cases they were not found to enhance resilience. Instead, they were associated with increased psychological distress and lower levels of resilience (Długosz, 2023; Kurapov et al., 2023; Xu et al., 2023) However, Xu et al. (2023) still counted self-distraction as a functional strategy, despite the fact that it was associated with increased distress. They also employ cognitive strategies, including acceptance, positive thinking, and finding meaning in their suffering, to normalize and mitigate the severity of their predicament (Posselt et al., 2018). Throughout the lengthy asylum process, refugees actively seek strategies and practices that contribute to their mental well-being. Our findings have pointed out the prevalent use of emotion-focused strategies, such as seeking emotional support, in the early stages of war, while problem-focused strategies, such as taking action to address the challenges, became more prominent as the conflict continued (Khraban, 2022; Xu et al., 2023). Furthermore, in line with our results (Oviedo et al., 2022; Rizzi et al., 2022; Talabi et al., 2022), building friendships with peers and adults who share similar ethnic backgrounds, as well as fostering positive relationships with the host community, positively impact the mental health of refugee seekers (Arakelyan and Ager, 2020). Social connections and participation in social activities serve as outlets for distraction, sharing experiences, and providing emotional and practical support (Posselt et al., 2018). Hence, the support and inclusiveness of host communities significantly contribute to improved psychological outcomes for refugees during periods of uncertainty and despair. Moreover, when discussing these results, it is crucial to remember that most of the refugees and IDPs are women (United Nations High Commissioner for Refugees, 2019). Reserachers have previously showed how men are more likely to adopt problem-focused strategies, while women are more inclined to concentrate on regulating their emotional reactions (emotion-focused coping) or to use avoidance (Billings and Moos, 1984; Stone and Neale, 1984; Endler and Parker, 1990). Specifically, our findings are consistent with coping research that suggest that support seeking is more common among women (Carver et al., 1989; Endler and Parker, 1990; Hobfoll et al., 1994). However, situations in which individuals have limited authority or where role expectations dictate behavior often offer little opportunity for exerting control and are more likely to be experienced by women. This suggests that gender differences in coping may be more a product of the specific settings then being primarily attributed to gender itself (Hobfoll et al., 1994). Further research is needed to investigate gender differences more in depth. Lastly, when analyzing protective factors and coping strategies, it is crucial to remember that culture plays a significant role in shaping coping strategies utilized by individuals in response to challenging situations, such as war and displacement.

Cultural factors, including beliefs, values, norms, and social support systems, influence how individuals perceive and interpret stressors and the available resources for coping. This was clearly demonstrated by the work of Chudzicka-Czupała et al. (2023a,b). Seguin and Roberts (2015) in their review described how cultural and religious beliefs shape the coping strategies of Eritrean refugees, as evidenced by Nordanger (2007). These strategies revolve around the belief that outward displays of grief can lead to physical illness and harm one’s relationship with God. Consequently, coping strategies focus on diverting thoughts, seeking distractions, and investing in the future. Similarly, Afghan residents, as observed by Eggerman and Panter-Brick (2010), employ coping strategies deeply rooted in cultural values. These strategies encompass faith, morals, perseverance, maintaining family unity, serving others and their country, and seeking social recognition and honor. The influence of cultural and religious beliefs on coping strategies was also evident among Iranian conflict-affected residents (Ebadi et al., 2009), female Darfuri students in Sudan (Badri et al., 2013), and Tibetan refugees (Ruwanpura et al., 2006; Sachs et al., 2008). These studies further underscore how cultural and religious beliefs shape the ways in which individuals cope with the challenges they face.

## 5. Conclusion

This systematic review highlighted the diverse range of coping strategies and risk and protective factors influencing the resilience of Ukrainian refugees and internally displaced persons (IDPs). Factors such as unresolved legal status, fear, poverty, and isolation contribute to the psychological decline of refugees and IDPs and hinder their social integration in host communities. Moreover, exposure to violence, both directly and indirectly, largely contribute to the risk of subsequent psychological disorders, as well as, witnessing violence and events that directly imperil individuals, families, or homes. On the other hand, strategies rooted in cultural values, faith, and maintaining a sense of belonging are commonly observed. The use of emotion-focused strategies, problem-focused strategies, and avoidance strategies varies in their effectiveness in enhancing resilience. Social connections, support networks, and inclusiveness significantly contribute to improved psychological outcomes for refugees and IDPs. As the war is still ongoing, longitudinal studies are warranted to examine the trajectory of coping strategies and resilience over time, particularly during different stages of displacement and resettlement. This can provide insights into the dynamic nature of coping and resilience and identify critical points for intervention and support. Furthermore, it is important to recognize the influence of culture in coping strategies. Further research is needed to explore the specific cultural and contextual factors that influence coping strategies and resilience of these populations. This can help develop a deeper understanding of their unique challenges and strengths and inform the development of culturally sensitive support and interventions that acknowledge individuals’ cultural backgrounds that can enhance the effectiveness of coping strategies and promote overall well-being.

To sum up, tailoring interventions to consider cultural values, beliefs, and social support systems can facilitate a more comprehensive and meaningful approach to supporting individuals in times of crisis. In addition, our findings highlighted how community-based interventions that promote social connections, cultural integration, and empowerment should be prioritized. Creating spaces for social interaction, cultural activities, and support groups can foster a sense of belonging and provide opportunities for sharing experiences and coping strategies. Collaborative and multidisciplinary care models should be implemented to address the complex needs of these populations. This may involve integrating mental health services with primary healthcare, social services, and community-based organizations to provide comprehensive support. According to World Economic Forum (2022), Ukraine has rapidly stepped up investment in and delivery of mental health services. Even before the war, Ukraine had embarked on an ambitious health reform process, including efforts to strengthen mental health services. This foundation has, by and large, enabled the wider mental health system to respond fairly quickly to the ongoing emergency. This is a noteworthy proof of Ukrainian population resilience that set an example for the entire WHO European Region facing similar challenges.

## Data availability statement

The raw data supporting the conclusions of this article will be made available by the authors, without undue reservation.

## Author contributions

DR: Funding acquisition, Writing – review & editing. GC: Conceptualization, Investigation, Methodology, Writing – original draft. ML: Investigation, Writing – original draft. MM: Writing – review & editing. CI: Conceptualization, Supervision, Validation, Writing – review & editing.
